# Down-regulation of miR-29c is a prognostic biomarker in acute myeloid leukemia and can reduce the sensitivity of leukemic cells to decitabine

**DOI:** 10.1186/s12935-019-0894-y

**Published:** 2019-07-10

**Authors:** Li-juan Tang, Guo-kang Sun, Ting-juan Zhang, De-hong Wu, Jing-dong Zhou, Bei-bei Ma, Zi-jun Xu, Xiang-mei Wen, Qin Chen, Dong-ming Yao, Jun Qian, Ji-chun Ma, Jiang Lin

**Affiliations:** 1 Laboratory Center, Affiliated People’s Hospital of Jiangsu University, 8 Dianli Rd., Zhenjiang, 212002 People’s Republic of China; 2 Department of Hematology, Affiliated People’s Hospital of Jiangsu University, 8 Dianli Rd., Zhenjiang, 212002 People’s Republic of China; 3 The Key Lab of Precision Diagnosis and Treatment of Zhenjiang City, Zhenjiang, Jiangsu People’s Republic of China; 4 Department of Hematology, The Third People’s Hospital of Kunshan City, 615 Zizhu Rd, Kunshan, 215300 People’s Republic of China

**Keywords:** MiR-29c expression, Acute myeloid leukemia, Prognostic, Decitabine

## Abstract

**Background:**

MicroRNA-29c (miR-29c) is abnormally expressed in several cancers and serves as an important predictor of tumor prognosis. Herein, we investigate the effects of abnormal miR-29c expression and analyze its clinical significance in acute myeloid leukemia (AML) patients. In addition, decitabine (DAC) has made great progress in the treatment of AML in recent years, but DAC resistance is still common phenomenon and the mechanism of resistance is still unclear. We further analyze the influences of miR-29c to leukemic cells treated with DAC.

**Methods:**

Real-time quantitative PCR (RQ-PCR) was carried out to detect miR-29c transcript level in 102 de novo AML patients and 25 normal controls. miR-29c/shRNA-29c were respectively transfected into K562 cells and HEL cells. Cell viability after transfection was detected by cell counting Kit-8 assays. Flow cytometry was used to detect apoptosis.

**Results:**

MiR-29c was significantly down-regulated in AML (*P *< 0.001). Low miR-29c expression was frequently observed in patients with poor karyotype and high risk (*P *= 0.006 and 0.013, respectively). Patients with low miR-29c expression had a markedly shorter overall survival (OS) than those with high miR-29c expression (*P *< 0.001). Multivariate analysis confirmed the independent prognostic value of low miR-29c expression in both the whole cohort as well as the cytogenetically normal AML (CN-AML) subset. Over-expression of miR-29c in K562 treated with DAC inhibited growth, while silencing of miR-29c in HEL promoted growth and inhibited apoptosis. MiR-29c overexpression decreased the half maximal inhibitory concentration (IC_50_) of DAC in K562, while miR-29c silencing increased the IC_50_ of DAC in HEL. The demethylation of the miR-29c promoter was associated with its up-regulated expression. Although miR-29c demethylation was also observed in DAC-resistant K562 (K562/DAC), miR-29c expression was down-regulated. MiR-29c transfection also promoted apoptosis and decreased the IC_50_ of DAC in K562/DAC cells.

**Conclusions:**

Our results suggest that miR-29c down-regulation may act as an independent prognostic biomarker in AML patients, and miR-29c over-expression can increase the sensitivity of both non-resistant and resistant of leukemic cells to DAC.

**Electronic supplementary material:**

The online version of this article (10.1186/s12935-019-0894-y) contains supplementary material, which is available to authorized users.

## Background

Acute myeloid leukemia (AML) is a malignant tumor of myeloid progenitor cells. It is characterized by the rapid growth of abnormal white blood cells in the bone marrow, which interferes with the production of normal blood cells. The pathogenesis involves the inhibition of cell differentiation, uncontrolled proliferation, abnormal apoptosis and so on [1, 2]. Despite recent advances in the molecular basis of leukemia and the use of new chemotherapy regimens, the overall outlook of AML remains poor. It has been well established that occurrence of leukemia is the result of different genetic changes, which ultimately leads to malignant transformation [3]. Epigenetic modifications such as DNA methylation and microRNA (miRNAs) expression also play a key role in the pathogenesis and progression of leukemia [4–9]. MicroRNAs are a class of endogenous, non-coding RNAs with regulatory functions that are about 20 to 25 nucleotides in length [10, 11]. Only a small fraction of the biological functions of miRNAs are currently elucidated. These miRNAs regulate cell growth, tissue differentiation, and thus are involved in development and disease during life [11, 12]. Over the last decade, many studies have confirmed that deregulated miRNAs activity can be responsible for hematologic malignancies [13, 14]. Some miRNAs may be used as potentially prognostic biomarkers. Alterations in the expression of miR-29, miR-125, miR-126, miR-142, miR-146 and miR-155 have been reported to play a role in the pathogenesis and progression of AML [15–17]. Let-7 family and the miRNA-34 family are known as tumor suppressor miRNAs in B cell lymphoma [18]. MiR-15, miR-16, miR 29a/b and miR-127 are also deregulated in chronic lymphocytic and AML [18, 19]. Gong et al. found intravenous injection of miR-29a/b/c could significantly ameliorate leukemia symptoms in AML model mice and revealed a key role for the miR-29 family in the development of AML [20]. MiR-29c, a member of microRNA-29 family, is located on chromosome lq32.2 [21–23]. Prominently, miR-29c has been shown to act as a tumor suppressor gene in various tumors and participate in the regulation of target genes of several important signaling pathways [24–28]. Butrym et al. found that miR-29c expression was up-regulated in older AML patients [29]. However, we got the opposite conclusion in this study. Therefore, this study further elucidates the roles of miR-29c in vitro with special attention to AML.

Additionally, DNA methylation changes are another pathological mechanism in leukemia progression. DNA hypomethylating agents (HMAs), including decitabine and azacitidine (AC), have achieved a considerable clinical role during these years [30, 31]. Studies have shown that HMAs exerts anti-tumor activity by re-activating methylation-silenced genes at low doses; whereas it plays a major role in cytotoxicity at high doses [32]. Moreover, responses in some patients are rather low, and these patients are often ephemeral-lived after HMAs failure. During the last few years, the molecular mechanism of anti-methylation therapy has not yet reached a consensus. Studies have shown that high MLL5 expression might increase the sensitivity to DAC in AML cells [33]. HMAs reduced the methylation level of the programmed death 1 (PD-1) promoter in AML cells, which was accompanied by worse survival [34]. Intriguingly, BCL2/adenovirus E1B 19 kDa interacting protein 3-like (BNIP3L) protein could promote apoptosis, but BNIP3L silencing slightly strengthens the apoptotic effect of decitabine in U937 cells [35]. These studies provide a new perspective for clinical and experimental research. Simultaneously, it is necessary to identify certain molecules that can distinguish between patient who will benefit from HMAs therapy and those who will not.

In the present study, we aimed to investigate the expression and methylation levels of miR-29c in leukemic cells. We further evaluated the clinical significance of deregulated miR-29c expression. To date, this study was the first to investigate whether miR-29c was associated with DAC resistance.

## Methods

### Study population

The present study included 102 newly diagnosed AML patients and 25 healthy donors. The patients were classified according to World Health Organization (WHO) criteria and French–American–British (FAB) classification. Table 1 listed the clinical characteristics of patients. BM mononuclear cells (BMMNCs) were extracted from BM specimens by gradient centrifugation (TBD Sciences, Tianjin, China). Mutations in FLT3, N/K-RAS, and U2AF1 were detected by high-resolution melting analysis [36–38].

**Table 1:** Correlation between *miR*-*29c* expression and patients’ parameters

Patient’s parameters	Status of *miR*-*29c* expression		
	Low (n = 51)	High (n = 51)	*P*
Sex, male/female	31/20	29/22	0.423
Median age, years (range)	54 (19–93)	58 (18–87)	0.968
Median WBC, × 10^9^/L (range)	12 (1.1–201)	8.1 (0.3–528)	0.517
Median hemoglobin, g/L (range)	76.5 (34–138)	71 (32–113)	0.202
Median platelets, × 10^9^/L (range)	42 (3–447)	37 (4–399)	0.196
BM blasts, % (range)	53 (6.5–94.5)	42.5 (1.0–97.5)	0.085
FAB classification			0.002
M0	1	0	
M1	5	4	
M2	23	24	
M3	1	14	
M4	14	6	
M5	6	3	
M6	1	0	
WHO classification			0.007
t (8;21)	7	5	
t (15;17)	1	13	
AML without maturation	4	4	
AML with maturation	18	18	
Acute myelomonocytic leukemia	15	7	
Acute monoblastic and monocytic leukemia	5	2	
Acute erythroid leukemia	1	0	
No data	0	2	
Risk classification			0.013
Low	8	20	
Intermediate	35	23	
High	8	4	
No data	0	4	
Karyotype			0.006
Normal	27	18	
t (8;21)	7	7	
t (15;17)	1	13	
11q23	1	0	
Complex	7	4	
Others	8	5	
No data	0	4	
Gene mutation*			
C-KIT (±)	2/47	1/47	1.000
FLT3 (±)	7/42	6/42	1.000
NPM1 (±)	5/44	5/43	1.000
C/EBPA (±)	7/42	5/43	0.759
N/K-RAS (±)	3/46	4/45	1.000
IDH1/2 (±)	3/46	3/40	0.704
DNMT3A (±)	6/43	3/40	0.494
U2AF1 (±)	1/48	4/39	0.332
CR (±)	33/17	26/21	0.305

*WBC* white blood cells, *FAB* French–American–British classification, *AML* acute myeloid leukemia, *CR* complete remission

+: positive; −: negative

* +: bi-allelicmutation; −: mono-allelic mutation or wild type

### RNA extraction, reverse transcription and real-time quantitative PCR (RQ-PCR)

According to the reagent specification, total RNA was isolated using Trizol reagent (Invitrogen, Carlsbad, CA, USA). RNA was reverse-transcribed to complementary DNA (cDNA) using miScript reverse transcription kit (Qiagen, Duesseldorf, Germany). The procedure of reverse transcription and RQ-PCR was conducted as previously reported [39, 40]. The forward primers for miR-29c were 5′-TAGCACCATTTGAAATCGGTTA-3′ and the reverse primers were universal primer provided by the manufacturer (miScript).

### DNA isolation, chemical modification, and Bisulfite sequencing PCR (BSP)

Genomic DNA was isolated and modified using genomic DNA Purification Kit according to the instructions (Gentra, Minneapolis, MN, USA). The primers for the methylated of miR-29c promoter were 5′-TAGTAGTGGTTGTTTGTTTTTTTGA-3′ (forward) and 5′-CCACTCTACTAAAAACTCCATCTCC-3′ (reverse). BSP conditions were conducted at 98 °C for10 s, 40 cycles for 10 s at 98 °C, 30 s at 65 °C, 30 s at 72 °C and followed by a final 7 min. at 72 °C. Then the company sequenced five independent clones from each sample (BGI Tech Solutions Co., Shanghai, China).

### Cell line, cell culture, DAC treatment, plasmid construction and transfection

Cell lines (including K562, HEL, THP-1, HL60, SHI-1 and NB4) were purchased from American Type Culture Collection (Manassas, VA, USA). The K562/DAC was constructed in our laboratory [41]. Cells were cultured in Roswell Park Memorial Institute-1640 (RPMI-1640, Wisent) containing 10% fetal calf serum (FBS, ExCellBio) and 100U/ml penicillin/streptom with 5% CO_2_ at 37 °C. The plasmids were designed and synthesized by GenePharma company (Shanghai, China). MiRNA (miR-29c) and shRNA (shRNA-29c) were transfected into cells using HiperFect (Qiagen). The stably transfected cells were selected by Geneticin (G418) or Blasticidin (Invivogen Company) and flow sorting (BD FACSAriall). Then cells were harvested and detected miR-29c expression by RQ-PCR.

### Cell viability assays

Cells (including K562-NC, K562-miR-29c, HEL-NC and shRNA-29c-HEL cells) were seeded at 3 × 10^3^ cells per well in 96-well plate containing complete culture solution and 1 μM DAC. After culturing for 0, 24 h, 48 h, and 72 h, each well added 10ul CCK-8 solution. The optical density (OD) was measured by microplate reader.

### Half maximal inhibitory concentration (IC_50_) detection

Cells (3 × 10^3^ cells/well) were seeded onto a 96-well plate containing complete culture solution and different concentration of DAC. The drug concentration was 0 μM, 0.125 μM, 0.25 μM, 0.5 μM, 1 μM, 2 μM successively. Cells were cultured for 48 h. OD value detection method was the same as cell viability assays.

### Cell apoptosis assays

Cells (5 × 10^5^ cells/well) were seeded onto a 6-well plate containing complete 1640 culture solution (without FBS solution). After 48 h, the apoptosis rate was detected by apoptosis detection kit (Annexin V PE/7-AAD, BD, 559763) and then analyzed by flow cytometry (BD FACSCalibur, San Jose, CA, USA).

### Statistical analysis

Data analysis was performed using SPSS 20.0 software and GraphPad Prism 5 software. Relative levels of miR-29c expression were calculated by 2^−ΔΔCT^ method. The categorical variables were analyzed using Chi square test and/or Fisher’s exact test. The diagnostic value of gene expression was analyzed using receiver operating characteristic curve (ROC curve) and area under the curve (AUC). Kaplan–Meier analysis and Cox regression analyses (univariate and multivariate) were used to analyze the survival. IC_50_ value was calculated by Probit regression analysis. Data analysis results for all experiments were statistically significant (*P *< 0.05, bilateral distribution).

## Results

### The level of miR-29c expression in normal controls and AML patients

**Fig. 1:**
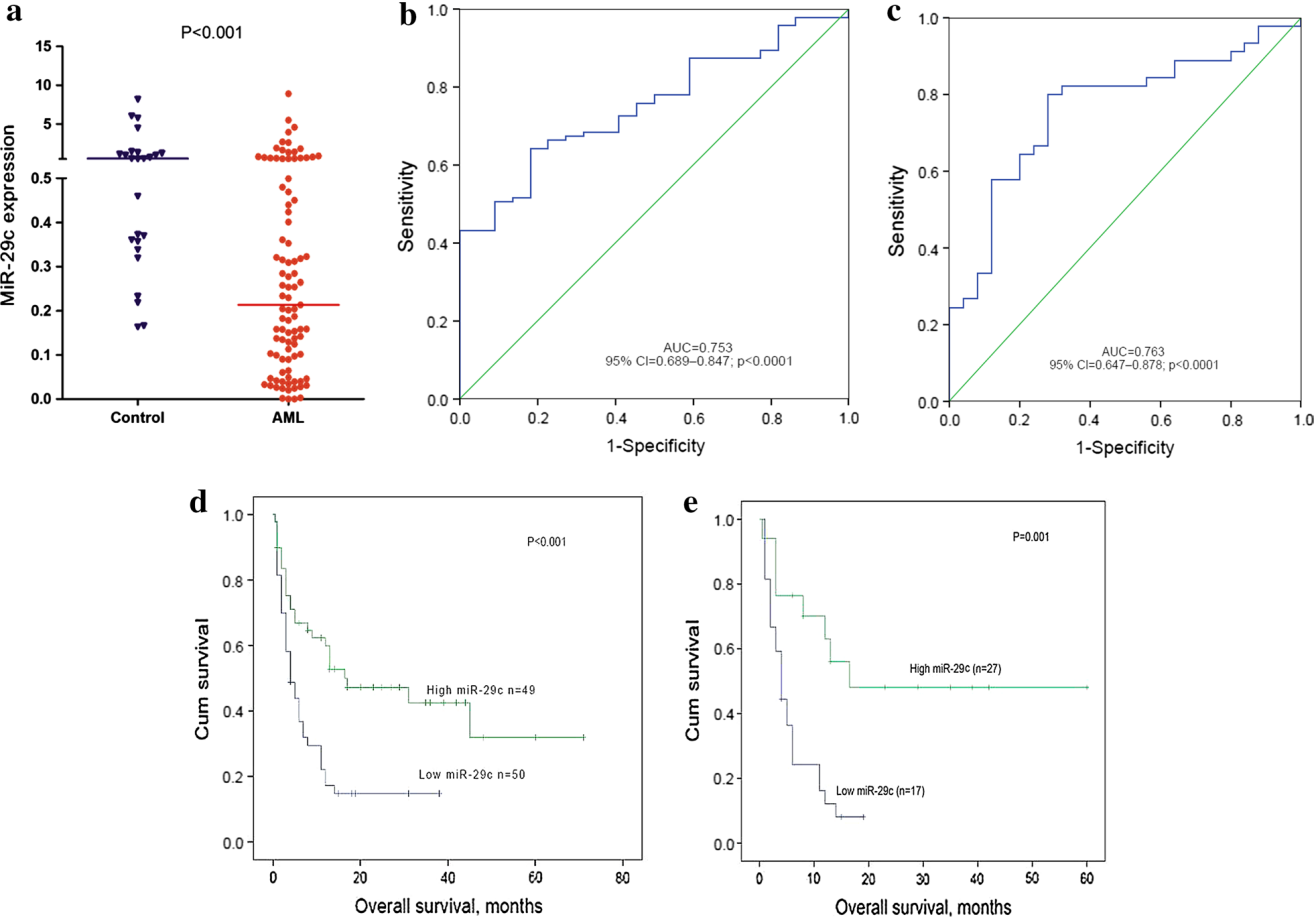
Expression level of miR-29c and impact of miR-29c expression on OS in AML patients. **a** The level of miR-29c expression in controls and AML patients by RQ-PCR. **b** Discriminative capacity of miR-29c expression by ROC curve analysis for miR-29c AML patients. **c** Discriminative capacity of miR-29c expression by ROC curve analysis for CN-AML patients. **d** Prognostic value of miR-29c expression in whole AML patients. **e** Prognostic value of miR-29c expression in CN-AML patients. Overall survival (OS) was analyzed between miR-29c high and miR-29c low groups

From RQ-PCR analysis, the median level of miR-29c expression was different in the normal control and AML groups (0.5703 and 0.2137, respectively). MiR-29c expression was significantly lower in AML compared with normal controls (*P *< 0.001) (Fig. 1a).

### Distinguishing capacity of MiR-29c expression

ROC curve was performed to determine the discriminative capacity of miR-29c expression. The AUC value of miR-29c was 0.753 (95% CI 0.689–0.874, *P *< 0.001) in all AML patients and 0.763 in CN-AML patients (95% CI 0.647–0.878, *P *< 0.001) (Fig. 1b, c), which suggested that miR-29c expression might serve as a potential biomarker in distinguishing AML patients from controls.

### Correlation between miR-29c expression and clinical characteristics in AML

To investigate the clinical relevance of miR-29c expression in AML, the whole patients were divided into two groups (low-expression and high-expression) by the cut off value of 0.187 (sensitivity 60%, specificity 80%) based on ROC curve. No significant differences were observed in age, BM blast cell percentage, gender, WBC (white blood cells), Hb (hemoglobin), PLT (blood platelet) and nine common gene mutations between the two groups (*P *> 0.05). However, obvious differences between the two groups were observed in karyotype and risk classification (*P *= 0.006 and 0.013, respectively). There was no correlation between the common gene mutations and miR-29c expression (*P *> 0.05).

### Effect of miR-29c expression on the outcome of AML patients

In order to explore the prognostic value of miR-29c in AML, survival data were obtained for 99 AML patients with follow-up data ranged from 1 to 70 months. Although miR-29c has no predicting value on complete remission (CR), patients with low miR-29c expression have markedly shorter OS time in both the whole AML cohort and the CN-AML subset (*P *< 0.001, Fig. 1d). Furthermore, similar results were observed in CN-AML patients (*P *< 0.001, Fig. 1e). Multivariate analysis showed the adverse effect of miR-29c low-expression on the outcome. Variables in the univariate analysis with *P *< 0.2 (WBC, age, and miR-29c expression, karyotype, K/N-RAS mutation, U2AF1 mutation) were included in the multivariate analysis. Importantly, the results showed that miR-29c might be an independent prognostic molecule affecting patients’ survival in all AML (*P *= 0.033) or CN-AML (*P *= 0.020, Tables 2 and 3).

**Table 2:** Univariate and multivariate analyses of prognostic factors for overall survival in whole-cohort AML patients

Prognostic factors	Univariate analysis		Multivariate analysis	
	Hazard ratio (95% CI)	P value	Hazard ratio (95% CI)	P value
Age (> 60/≤ 60 year)	2.840 (1.708–4.723)	< 0.001	1.381 (0.553–3.449)	0.489
WBC (≥ 30 × 10^9^/< 30 × 10^9^/L)	2.111 (1.424–3.127)	< 0.001	1.572 (0.420–4.132)	0.381
karyotype grouping	1.932 (1.567–2.383)	< 0.001	1.967 (1.375–2.841)	0.001
miR-29c expression	2.398 (1.427–4.032)	0.001	2.336 (1.026–5.319)	0.033
*K/N*-*RAS* mutation (±)	1.622 (0.840–3.131)	0.150	1.115 (0.147–8.467)	0.916
*U2AF1* mutation (±)	1.814 (0.732–4.500)	0.199	1.014 (0.492–5.806)	0.986
*FLT3* mutation (±)	1.109 (0.577–2.132)	0.757	–	–

+: positive; −: negative

**Table 3:** Univariate and multivariate analyses of prognostic factors for overall survival in CN-AML patients

Prognostic factors	Univariate analysis		Multivariate analysis	
	Hazard ratio (95% CI)	P value	Hazard ratio (95% CI)	P value
Age	2.080 (1.017–4.255)	0.045	1.125 (0.262–4.825)	0.874
WBC	1.522 (0.852–2.718)	0.049	1.174 (0.305–4.505)	0.816
miR-29c expression	3.546 (1.548–8.130)	0.003	6.897 (1.362 –34.483)	0.020
*K/N*-*RAS* mutation (±)	1.395 (0.548–3.550)	0.485	–	–
*FLT3* mutation (±)	1.185 (0.503–2.793)	0.697	–	–
*U2AF1* mutation (±)	1.181 (0.574–2.452)	0.651	–	–

+: positive; −: negative

### MiR-29c increased sensitivity to DAC and promoted apoptosis in leukemic cells

**Fig. 2:**
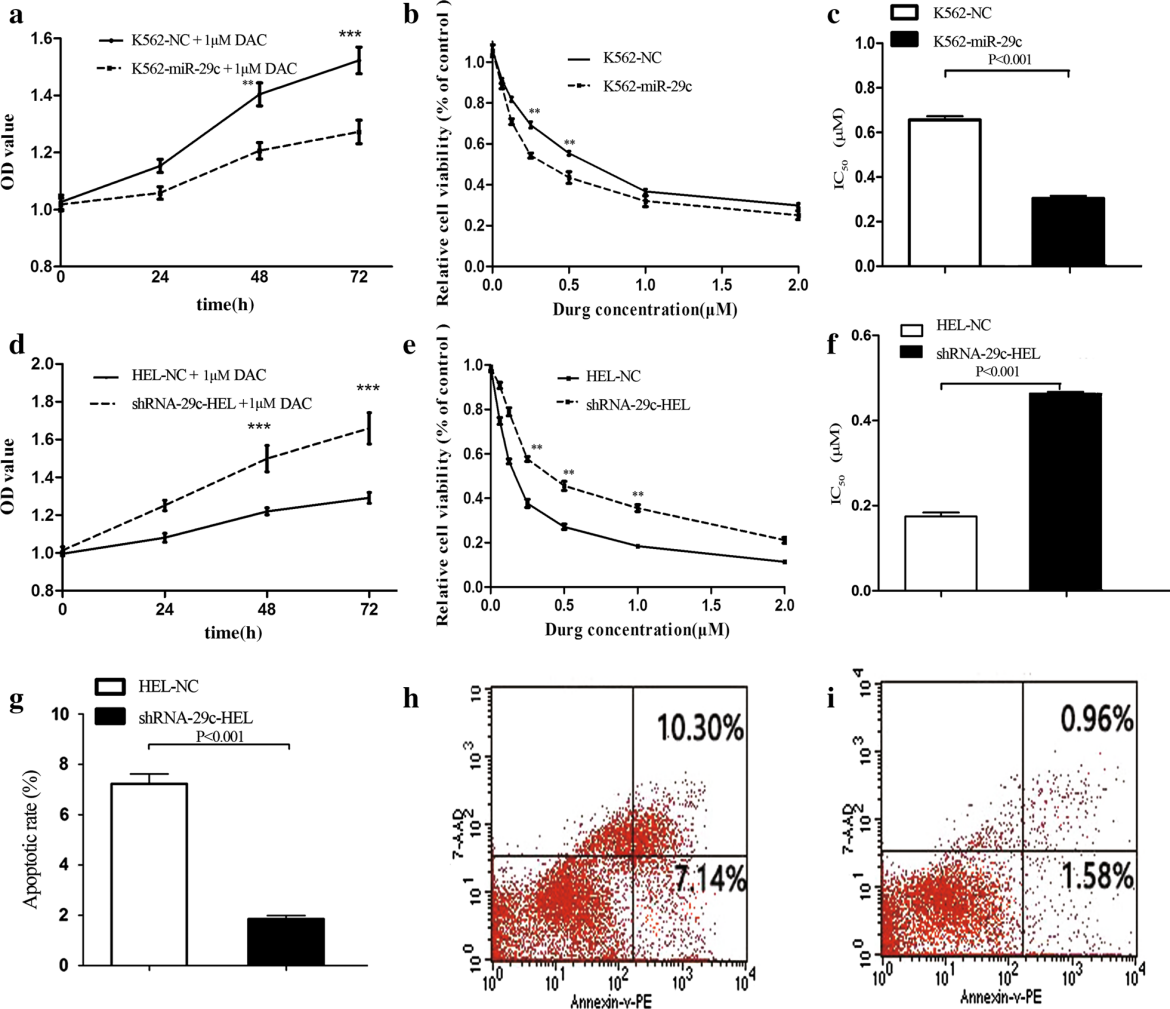
MiR-29c increased sensitivity to DAC and promoted apoptosis in leukemic cells. **a** CCK-8 Kit analysis the cell viability in K562-NC and K562-miR-29c cells. **b**, **c** Analysis the sensitive of DAC in K562-NC and K562-miR-29c cells by CCK-8 Kit. **d** CCK-8 Kit analysis the cell viability in HEL-NC and shRNA-29c-HEL cells. **e**, **f** Analysis the sensitive of DAC in HEL-NC and shRNA-29c-HEL cells by CCK-8 Kit. **g** The statistical analysis of Flow Cytometry about HEL-NC and shRNA-29c-HEL cells. **h** The apoptosis rate of HEL-NC cells was detected by Flow Cytometry. **i** The apoptosis rate of shRNA-29c-HEL cells was detected by Flow Cytometry. The IC_50_ was the half maximal inhibitory concentration

Before performing cell function experiments, we detected the expression levels of miR-29c in different leukemic cells, and found that miR-29c expression was decreased in K562, THP-1, HL60 and increased in HEL (Fig S1 in Additional file 1). Therefore, we selected HEL with the highest expression of miR-29c for gene gain-of-function. Additionally, we obtained DAC-resistant cells of K562. K562 cells were selected for gene loss-of-function. Increased expression of miR-29c in K562-miR-29c cells and decreased expression of miR-29c in shRNA-29c-HEL cells were confirmed by RQ-PCR (Fig. S2A, B in Additional file 1). The growth rate of K562-miR-29c was significantly lower than that of the K562-NC cells (*P *< 0.001, Fig. 2a, b). Similarly, the growth rate of shRNA-29c-HEL was obviously higher than that of the HEL-NC cells (*P *< 0.001, Fig. 2d, e). Moreover, miR-29c overexpression decreased the IC_50_ value of DAC in K562, while miR-29c silencing increased the IC_50_ of DAC in HEL (Fig. 2c, f). The overall apoptotic rate of shRNA-29c-HEL cells was significantly lower than that of HEL-NC cells (*P *< 0.001, Fig. 2g–i).

### Decreased expression of miR-29c might be involved in DAC resistance

**Fig. 3:**
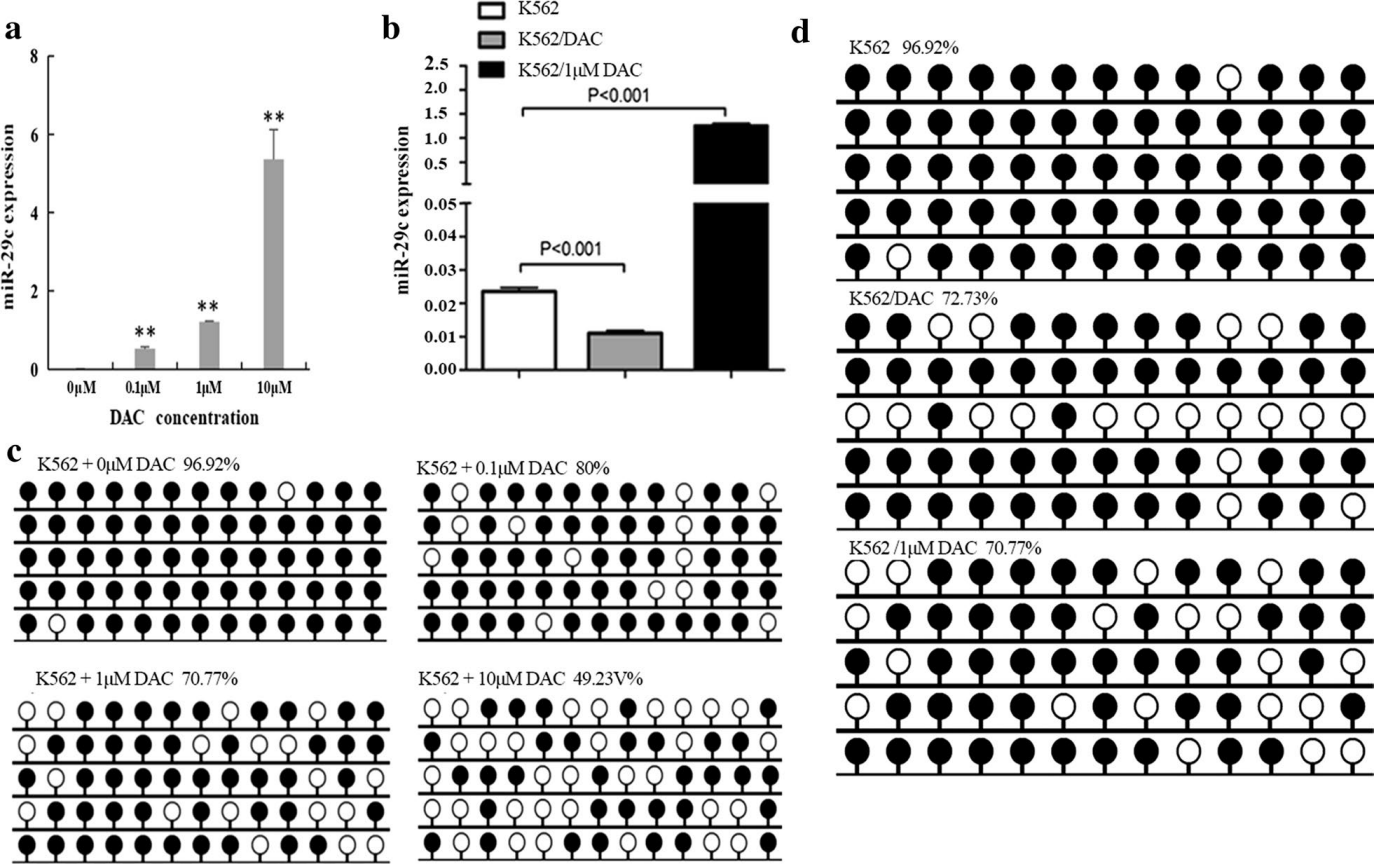
The expression and methylation level of miR-29c in K562 and K562/DAC. **a** The expression level of miR-29c in K562 under different DAC concentrations. **b** The expression level of miR-29c in DAC resistance cell (K562/DAC). **c** The methylation level of miR-29c in K562 under different DAC concentrations. **d** The methylation level of miR-29c in DAC resistance cell (K562/DAC). **a** K562 cell line. **b** K562/DAC cell. **c** K562 + 1 μM DAC. Methylation density of miR-29c in K562 and K562/DAC. White cycle: un-methylated CpG dinucleotide; black cycle: methylated CpG dinucleotide. **a**, **b** Controls (selected randomly); **c**, **d** un-methylated

To analyze the relationship of miR-29c expression and DAC resistance, the expression and promoter methylation of miR-29c were detected by RQ-PCR and BSP separately in K562 and K562/DAC cells. Results showed that the expression of miR-29c was up-regulated with increased DAC concentration in K562 cells (*P *< 0.01, Fig. 3a). The methylation density of the miR-29c promoter region was reduced (Fig. 3c). Although demethylation change was also observed in the miR-29c promoter region in K562/DAC cells (Fig. 3d), the level of miR-29c expression was down-regulated (Fig. 3b). This suggested DAC resistance was accompanied with miR-29c down-regulation.

### High miR-29c expression could increase the sensitivity of DAC-resistant cells to DAC

**Fig. 4:**
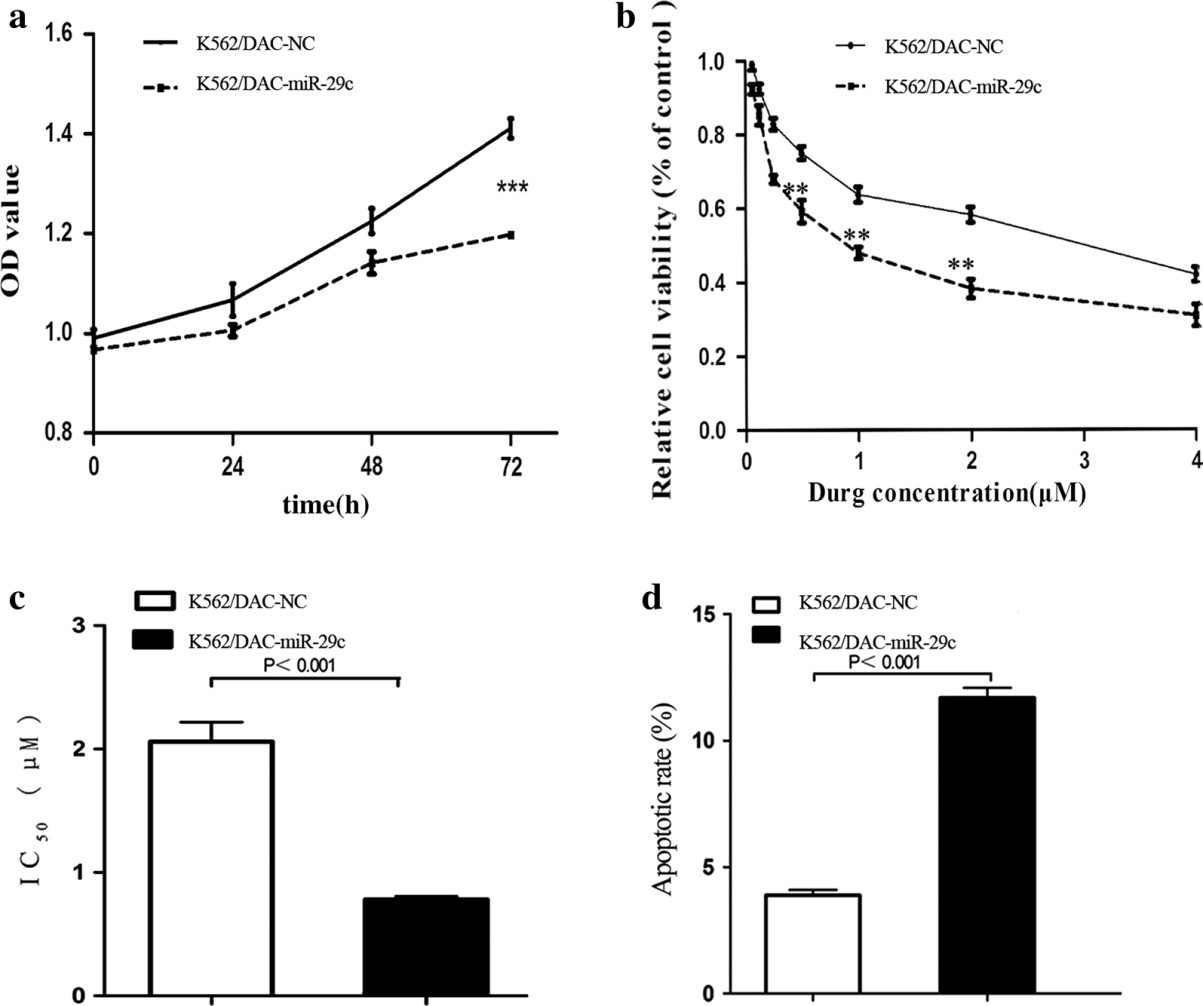
MiR-29c increased sensitivity to DAC and promoted apoptosis in DAC-resistant leukemic cells. **a** CCK-8 Kit analysis the cell viability in K562/DAC-NC and K562/DAC-miR-29c cells. **b**, **c** Analysis the sensitive of DAC in K562/DAC-NC and K562/DAC-miR-29c cells by CCK-8 Kit. **d** The statistical analysis of flow cytometry about K562/DAC-NC and K562/DAC-miR-29c cells cells. The IC_50_ was the half maximal inhibitory concentration

Increased expression of miR-29c in K562/DAC-miR-29c was confirmed by RQ-PCR (Fig. S3A in Additional file 1). Similarly, the viability rate of K562/DAC-miR-29c cells was significantly lower than that of the K562/DAC-NC cells (*P *< 0.001, Fig. 4a, b). The IC_50_ value of K562/DAC-miR-29c cells was significantly lower than that of the K562/DAC-NC cells (*P *< 0.001, Fig. 4c). Furthermore, the apoptosis assays showed that the apoptotic rate of K562/DAC-miR-29c cells was significantly higher than that of K562/DAC cells (*P *< 0.001, Fig. 4d, Fig. S3B, C in Additional file 1). The above results suggested that the miR-29c gene might alter the reactivity of DAC-resistant cells to DAC.

## Discussion

MiR-29c has been reported to be down-expressed in human solid tumors, such as breast cancer and lung cancer [42, 43]. Many studies have proved that miR-29c indeed acts as a tumor suppressor and miR-29c effects on cell proliferation, senescence, apoptosis and progression of cancer [44]. MiR-29c down-regulation was associated with poor survival in chronic lymphocytic leukemia, acute lymphoblastic leukemia, Burkitt lymphoma and chronic myeloid leukemia [45–48]. Conversely, Zhu et al. found that miR-29c was overexpressed in early stage non-small lung cancer (NSCLC), whereas the level of miR-29c expression did not relate with the OS of NSCLC patients [49]. Butrym et al. found that miR-29c was up-regulated and associated with poor prognosis in older AML patients [29]. However, our results showed that miR-29c expression was significantly down-regulated in AML and low expression of miR-29c was associated with shorter overall survival. We thought there were two pivotal reasons for our different results, as follow: (A) Subjects: Butrym et al. contained 95 patients (73 patients with primary leukemia and 22 patients with leukemia secondary to myelodysplastic or myeloproliferative syndrome). Our study included 102 newly diagnosed AML patients. Furthermore, the previous treatment of 22 patients with secondary leukemia was unknown. Namely, there were differences between the two studies’ subjects. (B) Treatment regimen: This is a vital factor affecting the prognosis of patients. Drug used by Butrym et al. was azacitidine. However, the patients were treated with conventional chemotherapy in our study. Additionally, our studies in vitro revealed that miR-29c over-expression could promote apoptosis and inhibited growth in leukemic cells treated with DAC, while miR-29c knock-down could reverse the effect.

In recent years, some miRNAs have been found to take part in chemotherapeutic drugs resistance, such as miR-138, miR-194, miR-137, etc. [40–52]. It has been reported that high miR-29c expression increased the sensitivity of non-small cell lung cancer (NSCLC) cells to the cisplatin [53]. Wang et al. found that miR-29c overexpression might contribute to the efficacy of cisplatin in gastric cancer treatment [54]. MiR-29c overexpression increased the sensibility of temozolomide-resistance cells in glioblastoma [55]. In vitro studies, miR-29c up-expression was accompanied with miR-29c demethylation during DAC treatment. It suggested that miR-29c expression might be regulated by its promoter methylation. Although the level of miR-29c expression was down-regulated, the promoter of miR-29c was demethylated in K562/DAC. This suggests that decreased expression of miR-29c may be involved in DAC resistance, which could be reversed by miR-29c over-expression.

## Conclusions

Taken together, our results indicate that down-regulation of miR-29c is a frequent event and predicts poor prognosis in de novo AML patients. MiR-29c overexpression can increase the sensitivity of leukemic cells to DAC and provides possible guidance for clinical DAC resistance.

## Additional file

**Additional file 1.** Additional figures.

## Data Availability

The datasets used and/or analyzed during the current study are available from the corresponding author on reasonable request.

## Abbreviations

DAC: decitabine, 5-Aza-2′-deoxycytidine
HMAs: hypomethylating agents
AC: azacitidine
RQ-PCR: real-time quantitative PCR
AML: acute myeloid leukemia
ROC curve: receiver operating characteristic curve
OS: overall survival
CR: complete remission
BSP: bisulfite sequencing PCR
CN-AML: cytogenetically normal AML
HEL-NC: HEL cells transduced with the plasmid of Neo-shNC
shRNA-29c-HEL: HEL cells transduced with the plasmid of homo-shRNA-29c
K562-NC: K562 cells transduced with the plasmid of Neo-shNC
K562-miR-29c: K562 cells transduced with the plasmid of homo-miR-29c
K562/DAC: K562 cells were resistant to DAC
K562/DAC-NC: K562/DAC cells transduced with the plasmid of Neo-shNC
K562/DAC-miR-29c: K562/DAC cells transduced with the plasmid of homo-miR-29c
IC_50_: the half maximal inhibitory concentration
